# The Preservation of the Limb in the Treatment of Sarcoma of Bone

**Published:** 1906-12

**Authors:** Charles A. Morton

**Affiliations:** Professor of Surgery in University College, Bristol; Surgeon to the Bristol General Hospital and the Royal Hospital for Sick Children and Women


### THE PRESERVATION OF THE LIMB IN THE TREATMENT OF SARCOMA OF BONE,

### WITH THE ACCOUNT OF A CASE IN WHICH THE UPPER HALF OF THE FIBULA WAS EXCISED FOR PERIOSTEAL SARCOMA.

BY

Charles A. Morton, F.R.C.S ,

*Professor of Surgery in University College, Bristol; Surgeon to the Bristol General Hospital and the Royal Hospital for Sick Children and Women.*

---

There is no doubt that periosteal sarcoma is a very malignant form of sarcoma; it tends to recur in the stump, or the neighbourhood of the stump, after amputation, and in spite of amputation metastatic growths, especially in the lung, are very likely to occur. It is more malignant when attacking some bones than others. In the femur and humerus the tendency to recurrence is terribly great. Nearly all British surgeons advocate amputation for periosteal sarcoma, and some urge higher amputation than is usually done at the present time for periosteal sarcoma of the femur and humerus—hip-joint amputation for all growths affecting the femur, and Berger's amputation rather than amputation at the shoulder-joint for such growths of the humerus in its upper part. On the other hand, the generally-recognised treatment of sarcoma of a muscle is not amputation but excision of the growth, though I know one surgeon has consistently advocated amputation for such growths, because he considers that they are as malignant as periosteal sarcomas of bone, or at any rate of certain bones. But the majority of British surgeons are not consistent about this, for there is really no reason why we should amputate a limb for a periosteal sarcoma of the tibia and excise a round-celled sarcoma of the tibial muscles.

In a paper read before the Bristol Medico-Chirurgical Society in December, 1904, I advocated the preservation of the limb in certain cases of periosteal sarcoma. Since then I find some continental surgeons have adopted this practice for several years. In the *Lancet* (vol. i., 1906, p. 82), Professor Goldmann, writing on the "Conservative Operations for Malignant Growths of Long Bones," says: "Judging from various publications following upon Mikulicz's paper of 1895, it appears that our view as regards the operative treatment of malignant growths of long bones has undergone a change. Whereas it has hitherto generally been accepted that, especially in cases of periosteal sarcoma, amputation or removal of the whole of the diseased bone, is indicated, we now consider conservative treatment, consisting in excision of the tumour only, justifiable, and more so when an early stage of the growth is diagnosed." Professor Goldmann refers to two very encouraging cases in which he excised portions of the tibia for periosteal sarcoma, and there was no recurrence six years later in either. In one of these cases a considerable portion of the tibia was excised, and the very ingenious plan adopted of dividing the upper end of the fibula and inserting its pointed end into a hole bored in the upper end of the tibia. Ten weeks after the operation the patient was able to support the full weight of the body on the limb, and in the course of two years radiographs showed that the fibula had become almost as thick as the tibia.[1]

Gross in 1879[2] published a paper giving statistics of the course of various forms of sarcoma of bone. Unfortunately these statistics are not of consecutive cases, but they are nevertheless of considerable value. I fear consecutive cases would give even a worse prognosis. Gross shows that, taking all kinds of periosteal sarcomata together (spindle-celled, round-celled and osteoid), in 37 cases followed up after amputation, metastases developed in two-thirds, and in rather less than one-half local recurrence took place. This shows how liable the patient is, not only to local recurrence after amputation,

1 Mikulicz, "encouraged by Von Bergmann's and Von Bramann's success in resecting the tibia, performed resection, instead of a more radical operation, for a periosteal spindle-cell sarcoma, involving the lower third of the femur." He removed eight inches of the femur, and then sawed off the cartilaginous surface of the tibia, and inserted the lower end of the femur in a hole bored in the lower end of the tibia. "Wiesenger also recently resected three case successfully."—*System of Practical Surgery*, by Von Bergmann, Von Bruns and Von Mikulicz. Trans. by W. T. Bull, vol. iii., p. 557.

2 *Am. J. M. Sc.*, 1879, lxxviii. 17, 338.

but even to a greater degree to secondary distant growths, though possibly if these growths had been excised rather than the limb amputated local recurrence might have been greater, yet still two-thirds would have died from metastases.

Wyeth's statistics are even more striking.[1] He collected 51 cases of fatal recurrence of sarcoma of the bones[2] of the lower limb after amputation at the hip joint, and of these no less than 36 had metastases without local recurrence.

The tendency to metastases rather than to local recurrence in periosteal sarcoma of the *leg bones* is further shown by the statistics of cases given in Mr. Butlin's *Operative Surgery of Malignant Disease.* Out of 21 cases which recovered after amputation, 16 died of metastases, and only 3 of these had local recurrence. On the other hand, Mr. Butlin has shown (*op. cit.*) that the tendency to recurrence in the limb after amputation, as well as to metastases, in periosteal sarcoma of the femur and humerus is very terrible, and it is for these cases he advises hip-joint amputation, even if the growth affects the distal end of the femur, and Berger's amputation when the growth affects the upper portion of the humerus. But he admits that amputation at the hip-joint has given no better results than amputation through the shaft, and holds out no prospect of permanent success. Although amputation at the hip-joint, as Mr. Butlin points out, has not now the very high mortality it had years ago, and the mortality of Berger's operation has fallen to 6 per cent. in 32 consecutive cases, and although it is with the greatest hesitation that I should venture to form any opinion at variance with Mr. Butlin's on such a subject as the treatment of malignant tumours of bone, yet I do venture to say that if the outlook is so terrible even with high amputation, my own feeling would be that it might be wiser not to sacrifice the limb, if we could excise a small, well-encapsuled growth, even on the femur or humerus, and leave a useful limb.

I must admit that every case of local recurrence is a possible source of secondary growth in the lung; but if we have the

[1] *Ann. Surg.*, 1901, xxxiv. 375.

[2] It is almost, but perhaps not quite, certain from his paper they were all sarcomata of bone. He refers in his paper to it as a "Study of Sarcoma of the Long Bones."

patient under frequent observation after excision of the growth, and can re-excise or amputate at once if there are signs of recurrence, we diminish this risk considerably. And we must remember that whether we amputate or excise the growth, in no less than two-thirds of the cases (and a larger proportion in the leg bones) metastases will occur.

I have dissected three cases of periosteal sarcomata of the bones, and in all the cases I found the growth well encapsuled; and Gross (*loc. cit.*) has shown that this is so in no less than half all periosteal sarcomata. I should only advise the excision of the non-infiltrating growths. If the growth was found to be infiltrating the surrounding parts, at any rate widely, I should amputate well above it. All muscles arising from the capsule of the growth should be cut at a distance from it, and I should always excise at least an inch of healthy bone above and below it, and I should, directly the portion of bone with the growth was excised, get a longitudinal section of it made to see if the growth had extended farther up the bone in the interior than on the exterior, and if so further bone should be removed. The whole fibula might be excised. Professor Goldmann (*loc. cit.*) has shown how much of the tibia may be excised. I should not hesitate to remove four inches of the femur, or even more of the humerus, and I should of course screw or wire the ends together. I think most persons would prefer a much shortened limb to an artificial one. Of course if such removal involved the loss of the most actively growing epiphyses of the bone in a young person, the late shortening, in addition to the immediate shortening, might be too great to make excision practicable. Large portions of either bone of the forearm could be excised, and after excision of a portion of the radius (as has been done in excision for myeloid sarcoma at the present day), it would be well to shorten the ulna to the same extent, and perhaps the tendons as well.

I have already stated that those surgeons who advise amputation for every periosteal sarcoma, and only excision of sarcomata of the soft parts, are not consistent. Granting that periosteal sarcoma of the femur and tibia are more malignant than any other form of sarcoma, we have no statistics showing that periosteal sarcomata of the other long bones are more apt to recur *in oco* than sarcomata of muscles, fascia, joints and nerves. Mr. Butlin[1] has shown how malignant sarcomata of muscles are, and he consistently advises amputation for them. To Mr. Butlin's list of collected cases I can add one of my own of excision of sarcoma of the muscles of the forearm. There was no local recurrence, but the man died with a large growth in his chest a year later; and in another case of sarcoma of muscle and fascia at the back of the neck I have had to operate again and again for recurrence. Wyeth[2] records a case of sarcoma of the internal popliteal nerve, which twice recurred in the thigh after excision; finally the patient died with recurrence in the lungs twelve months after amputation at the hip, the stump remaining free. Howard Marsh[3] records a case of sarcoma of the synovial membrane of the knee, which recurred five times in six years after excision. Edington[4] records a case of death from secondary growths in the lungs and other parts fifteen months after excision of the scapula for spindle-celled sarcoma of the infra-spinatus muscle.

As it is generally recognised that endosteal sarcomata as a class are distinctly less malignant than periosteal, and that myeloid sarcoma usually does not recur *in loco* after excision, and only rarely leads to metastatic growths,[5] I need not urge excision of such growths rather than amputation. Indeed, the excision of myeloid sarcoma is now the rule of surgery. Some surgeons would also excise the more malignant endosteal growths. With these, however, the same precautions should be taken as to removal well free of the growths, I have specified with regard to periosteal sarcoma. In many cases we cannot tell whether a growth is endosteal or periosteal until we have excised it, and a myeloid sarcoma cannot often be with certainty diagnosed, at any rate until we have cut into the tumour.[6]

[1] *Operative Surgery of Malignant Disease*, 2nd Edition, 1900.

[2] *Ann. Surg.*, 1901, xxxiv. 375.

[3] *Tr. Clin. Soc. Lond.*, 1899, xxxii. 20. [4] *Brit. M. J.*, 1901, i. 954.

[5] Mr. Bland Sutton has proposed to call such tumours no longer sarcoma but myeloma.

[6] Gross estimated the frequency of myeloid sarcoma as 70 per cent. of all endosteal sarcomata, and out of eight cases of central sarcoma of the upper end of the tibia, referred to by Butlin and Colby (*St. Barth. Hosp. Rep.*, 1895, xxxi. 35), seven were myeloid.

I shall here record my own case of excision of half the fibula for sarcoma, and refer to another case of the same kind, published by Mr. Bland Sutton, and also refer to two cases of myeloid sarcoma of the head of the tibia in which several years ago I excised the portion of bone containing the growth, but saved the limb. As I have already stated, it has for some years now been the rule to excise myeloid sarcomata, but until I published these two cases[1] such growths in the main bones of the lower limb had not been excised. My first case has shown what a useful limb may result from such excision. The condition of the limb soon after the excision is fully described and illustrated in my original paper (nearly four inches of bone were excised), and I am glad to say the limb is still quite as useful to the patient, who is daily engaged in laborious work, and when I examined him last, six years after the excision, there were no signs of any recurrence. The other patient had a useful limb after the operation, but I cannot trace her now.

**Case of Excision of Half the Fibula for Periosteal Sarcoma.**— The patient, a woman, E. B., aged 27, was sent to me at the end of September, 1904, by Dr. James Young, with a tumour in the calf. It had been first noticed in May, when she was under the care of another doctor. It was hard and the size of a cocoanut, and was attached to the upper part of the right fibula, and projected backwards into the calf, and also outwards. At the upper part of the anterior interosseous space a smooth boss projected from the larger mass. A skiagram showed irregular bony projections from the shaft of the fibula, in the region of the growth. This made it quite certain that it was not merely a tumour attached to the bone, but a periosteal sarcoma. The dorsalis pedis artery could not be felt in the foot of the limb in which the tumour was situated, but could plainly on the other side. The posterior tibial artery could not be felt on either side.

I determined, if possible, to remove the growth, with the upper end of the fibula, rather than amputate the leg, but I did not feel sure that this could be done, for I thought

[1] *Brit. M. J.*, 1898, ii. 228.

the growth might have involved both the anterior and posterior tibial arteries, as well as the peroneal, and that by its removal serious risk of gangrene might be incurred. However, only the anterior tibial artery had to be tied. It was impossible to dissect out the boss of growth in the upper part of the anterior interosseous space without injury to this vessel. The external popliteal nerve, which ran in a groove on the tumour, I was able to preserve, and its two branches were not injured. Nearly the whole, if not the whole, of the peroneus longus had of course to be removed, and the upper portion of several other muscles, and the fibular attachment of the soleus, and the greater part of the fibular attachment of the tibialis posticus. The biceps and external lateral ligament of the knee joint had also to be severed, for the whole of the upper end of the fibula was removed. The bone was divided about its middle, well below the lowest level of the tumour.

The tumour was completely encapsuled in every direction except at its attachment to the bone. It contained a large amount of cartilage, and spicules of bone radiated into it from the shaft of the fibula. A microscopic section showed the sarcoma cells to be of irregular shape, and areas of sarcoma were mixed with areas of cartilage in the same section. The growth did not completely surround the shaft.

The wound, which was a very extensive one, was drained for forty-eight hours, and ran an aseptic course. The limb was kept on a back splint, with foot-piece, for a month after the operation, and then she began to walk on it. There was then a tendency for the foot to invert, and for some time she used a lateral splint at night. This tendency had by December quite passed off, and she then walked with only a slight limp, and without the aid of a stick. She could not evert the foot, nor to any extent invert it, but the flexors and extensors acted well, and the foot remained in normal position both when at rest and during walking. At this time I showed the patient and specimen at the Bristol Medico-Chirurgical Society. She has since had two recurrences in the leg; the first was excised eight months after the original operation, and the second six months later (ten months ago). At the first operation for recurrence, the anterior tibial nerve had to be sacrificed, and she got paralysis of the extensors, but the power returned, and she could walk quite well. At the second operation for recurrence, part of the posterior tibial artery had to be removed, and also part of the external popliteal nerve, and paralysis of the extensors now persists, but she can walk for several miles on the limb without aid of any kind. Last summer I treated her with injections of Coley's fluid to try and prevent further recurrence. She is now (November, 1906, two years after the removal of the growth) free from recurrence.

What the prognosis is for periosteal sarcoma of the fibula treated by amputation it is difficult to say. Mr. Bland Sutton writing in 1896[1] says he could only find the complete records of 3 such cases; but I have already referred to Mr. Butlin's statistics of 21 cases, taking both bones together, with 16 deaths from metastases after amputation. The great majority of these were in the tibia, but there is no reason to think that growths in the fibula would be more exempt from metastases after operation.[2] The result so far, then, in my case is not discouraging; nor, even in Mr. Bland Sutton's case (of which details will presently be given) though the patient eventually died of metastases, was the result worse than in the majority of cases of periosteal sarcoma of the tibia treated by amputation. Coley has lately proposed[3] to treat operable sarcomas of bone by the injection of his fluid in order to save the limb. He has collected twelve cases of sarcoma of the extremities, three personal cases, and nine reported by other surgeons, in which the use of the toxines rendered amputation unnecessary. Of these, eight were free from recurrence three to six years. In all the cases but one the tumour was of bony origin, and in all amputation had been seriously considered, but it seemed justifiable to give the toxines a trial before resorting to it. I must say I should rather myself excise such growths where excision was practicable, but as a means of preventing recurrence Coley's fluid seems to me well worth trying, for if it can cause the disappearance of the tumour, how much more likely is it to destroy the few cells of the tumour

[1] *Brit. M. J.*, 1896, i. 1087.

[2] Mr. Butlin in *Operative Surgery of Malignant Disease* says they grow faster than similar growths in the tibia. [3] *Am. J. M. Sc.*, 1906, cxxxi. 375.

lying in the tissues, which eventually grow into a "recurrence." The fluid has been largely used in this way by Coley.[1]

After operating on my own case I found the record of a similar case by Mr. Bland Sutton.[2] The tumour was a round-celled sarcoma, and was of considerable size. Mr. Sutton had to divide the musculo-cutaneous nerve, but united it at the end of the operation. The anterior tibial nerve, as well as the anterior tibial artery, had to be sacrified, and this, of course, led to paralysis of the extensors; but by means of an "orthopædic arrangement" the patient walked "excellently." In the edition of his book on tumours, published in 1903, he has given the after progress of the case, and it is as follows: The growth recurred in the scar eighteen months after excision, and was again removed; but a more extensive recurrence took place six months later, and amputation had to be done. She died two and a half years after the first operation with signs of growth in the lungs. Mr. Bland Sutton has not proposed to extend the treatment adopted in this case to periosteal sarcomas on other bones. He decided not to amputate for periosteal sarcoma of the fibula because he considered it was not as malignant a form of sarcoma as periosteal sarcoma in some other bones.

Since advocating excision of certain periosteal sarcomata at the meeting of the Bristol Medico-Chirurgical Society in December, 1904, I have performed Berger's interscapulo-thoracic amputation for sarcoma of the upper end of the humerus. I did so because, as it was evident from the size of the growth and the proximity to the shoulder-joint amputation must be performed there, I did not see that the scapula and outer half of the clavicle would be any good to the patient, and the mortality of Berger's operation has not been great in recent cases. Therefore I felt inclined to sacrifice the shoulder girdle.[3] The growth was very large, and was broken up by hemorrhage. She had no local recurrence, but died from pulmonary disease, possibly due to metastases, a year after the operation.

[1] Vide *Bristol M.-Chir. J.*, 1906, xxiv. 148. [2] *Brit. M. J.*, 1896, i. 1087.

[3] An examination after amputation showed that amputation at the shoulder-joint would have divided the muscles and ligaments too near the growth.

There is one question I ought to refer to before concluding this paper, and that is—Whether instead of excising the portion of bone containing a myeloid sarcoma we might simply scrape the growth out of the bone? This has been done in several cases, and without recurrence when the patient was seen about two years after the operation, and in one case eight years after; but it is not always successful in preventing recurrence, for Mr. Butlin[1] mentions one case which recurred after scraping,[2] and amputation was performed two years after the first operation. Moreover, in two cases a sinus has remained for two years[3] after a myeloid sarcoma had been scraped out of the bone. I have also had a case myself which impressed me very much with the difficulty of getting the cavity in the bone to fill up.

A man was under my care in the Hospital with enlargement of the upper end of the tibia, the nature of which was doubtful, and on exploration I found the head of the tibia occupied by a mass of very soft, friable, yellow material, which could be easily turned out by the finger. It looked more like a degenerate gumma than anything else I had seen, but microscopic section of a little piece of firmer tissue than the rest, showed a structure so full of giant cells, it was probably a myeloid sarcoma which had undergone fatty degeneration. However, although the case was of great interest from the point of diagnosis, I refer to it here because the cavity, which was large, took so long to fill up, and a sinus persisted for so many months and prevented the patient returning to work (and may still persist, for he has left Bristol) that I wished I had resected the upper end of the tibia, for I am sure he would have preferred a shortened limb to one with a persistent sinus. Probably in cases in which we do scrape out a myeloid sarcoma it would be well to fill the cavity with decalcified bone chips to form a medium for the growth of new tissue, or better still to adopt the method of filling the cavity with wax and iodoform.

[1] *Operative Surgery of Malignant Disease*, 1900.

[2] Mr. Sheild also (*Lancet*, 1901, i. 97) refers to a case of recurrence after enucleation of myeloid sarcoma.

[3] Butlin, *Op. cit.*, and Hinds, *Brit. M. J.*, 1898, i. 555.

